# Neuropsychiatrische Manifestationen geschlechtschromosomaler Aberrationen – klinische und therapeutische Aspekte der neurologisch-psychiatrischen Versorgung

**DOI:** 10.1007/s00115-026-01977-0

**Published:** 2026-06-02

**Authors:** Heiko Paland, Alexandra Philipsen, Eva C. Schulte

**Affiliations:** 1 Klinik und Poliklinik für Psychiatrie und Psychotherapie, Universitätsklinikum Hamburg-Eppendorf (UKE), Martinistr. 52, 20246 Hamburg, Deutschland; 2 Klinik und Poliklinik für Psychiatrie und Psychotherapie, Universitätsklinikum Bonn, Bonn, Deutschland; 3 Institut für Humangenetik, Universitätsklinikum Bonn, Bonn, Deutschland; 4 Institut für Psychiatrische Phänomik und Genomik (IPPG), LMU Klinikum, LMU München, München, Deutschland; 5 DZPG (German Center for Mental Health), Partner Site, München/Augsburg, München, Deutschland

**Keywords:** Neurodevelopmentale Störungen, Psychische Störungen, Autismusspektrumstörung, Aufmerksamkeitsdefizit‑/Hyperaktivitätsstörung, Seltene Erkrankungen, Neurodevelopmental disorders, Mental health disorders, Autism spectrum disorder, Attention deficit disorder with hyperactivity, Rare diseases

## Abstract

**Hintergrund:**

Geschlechtschromosomale Aberrationen betreffen etwa 1,5 pro 1000 Individuen, wobei somatisch wenig ausgeprägte Phänotypen unerkannt bleiben können. Auch bei bekannter Diagnose gilt es, das erhöhte Risiko neuropsychiatrischer Manifestationen spezifisch in der psychiatrischen Versorgung zu adressieren.

**Methodik:**

Die narrative Übersicht analysiert aktuelle Literatur zu neuropsychiatrischen Manifestationen geschlechtschromosomaler Syndrome mit Fokus auf die vier häufigsten Formen (Klinefelter‑, Turner‑, XYY- und Triple-X-Syndrom).

**Ergebnisse:**

Geschlechtschromosomale Aberrationen zeigen charakteristische neuropsychiatrische Manifestationen. Häufige psychiatrische Manifestationen umfassen Aufmerksamkeitsdefizit‑/Hyperaktivitätsstörung, Autismusspektrumstörungen, Angststörungen, affektive und psychotische Störungen. Kognitive Beeinträchtigungen betreffen die Mehrheit der Betroffenen und manifestieren sich in sprachlichen Entwicklungsstörungen, exekutiven Dysfunktionen oder in visuell-räumlichen Defiziten. Schwere intellektuelle Beeinträchtigungen sind selten (< 5–10 %).

**Schlussfolgerung:**

Erhöhte klinische Aufmerksamkeit für neuropsychiatrische Manifestationen geschlechtschromosomaler Aberrationen ermöglicht eine frühzeitigere Diagnosestellung und Zugang zu entwicklungsfördernden, psycho(pharmako)logischen und endokrinologischen Behandlungsansätzen zur Verbesserung der Lebensqualität und gesellschaftlichen Teilhabe Betroffener.

**Zusatzmaterial online:**

Die Online-Version dieses Beitrags (10.1007/s00115-026-01977-0) enthält umfangreiches Zusatzmaterial.

## Abstract (en)

**Background:**

Sex chromosome aberrations affect approximately 1.5 per 1000 individuals, although somatically inconspicuous phenotypes can remain undetected. Even when diagnosed, the elevated risk of neuropsychiatric manifestations needs to be specifically addressed in psychiatric care.

**Methods:**

This narrative review analyzes the current literature on neuropsychiatric manifestations of sex chromosome syndromes, focusing on the four most common forms (Klinefelter, Turner, XYY and triple‑X syndromes).

**Results:**

Sex chromosome aberrations show characteristic neuropsychiatric manifestations. Frequent psychiatric manifestations include attention deficit hyperactivity disorder, autism spectrum disorders, anxiety disorders, affective and psychotic disorders. Cognitive impairments affect the majority of individuals and are manifested in speech developmental disorders, executive dysfunctions or visuospatial deficits. Severe intellectual impairments are rare (< 5–10%).

**Conclusion:**

Heightened clinical attention to neuropsychiatric manifestations of sex chromosome aberrations facilitates earlier diagnosis and access to developmental, psycho(pharmaco)logical and endocrinological treatment approaches to improve the quality of life and social participation of those affected.

## Einführung

Geschlechtschromosomale Aberrationen (GCA), insbesondere das Turner-Syndrom (TS; 45,X), Klinefelter-Syndrom (KS; 47,XXY), XYY-Syndrom (47,XYY) und Triple-X-Syndrom (47,XXX), betreffen circa 1,5/1000 Individuen [23]. Hochgerechnet ergäbe dies eine geschätzte Zahl von ca. 150.000 Betroffenen in Deutschland. Klinisch werden insbesondere GCA mit auffälligem somatischem Phänotyp erkannt [2].

Basierend auf skandinavischen Studienergebnissen [1, 2] muss davon ausgegangen werden, dass ein größerer Anteil von GCA (insbesondere KS, XYY, XXX) bisher nicht diagnostiziert ist und eine relevante Patientengruppe in der Erwachsenenpsychiatrie darstellt. Dadurch fehlt den Betroffenen Frühförderung und eine auf GCA abgestimmte Behandlung somatischer und psychiatrischer Manifestationen.

Kardinalsymptome wie Kleinwuchs beim TS, Hochwuchs und Hypogonadismus beim KS sowie variabel präsente Merkmale basieren auf historischen Fallserien, in denen Schwerbetroffene im Vergleich zur Gesamtbevölkerung überrepräsentiert sind [9, 25].

Populationsbasierte Studien zeigen systematisch erhöhte Risiken für neurodevelopmentale und psychiatrische Manifestationen, insbesondere Autismusspektrumstörungen (ASS), Aufmerksamkeitsdefizit‑/Hyperaktivitätsstörung (ADHS), Schizophreniespektrumstörungen (SSS) und affektive Störungen [14, 23]. Beachtlich ist, dass ca. 85 % aller Menschen mit GCA-Kriterien mindestens eine psychiatrische Diagnose nach ICD-10 (International Statistical Classification of Diseases and Related Health Problems 10) erfüllen [23]. Komplementär hierzu zeigen sich neurokognitive Signaturen wie spezifische IQ-Muster, Sprachdefizite, exekutive Dysfunktionen, Beeinträchtigungen der Verarbeitungsgeschwindigkeit, motorische Koordinationsstörungen und Beeinträchtigungen sozialer Kognition [15, 29].

Neuropsychiatrische Muster von GCA sollten in psychiatrisch-differenzialdiagnostische Überlegungen einfließen und die Veranlassung weiterführender genetischer Diagnostik in Verdachtsfällen sollte nicht allein in der Hand pädiatrischer oder humangenetischer Kollegen liegen. Ziel dieser Übersicht ist es, psychiatrisch Tätigen einen praxisorientierten Überblick über neuropsychiatrische Manifestationen der vier häufigsten GCA zu geben, von der differenzialdiagnostischen Einordnung bei bislang undiagnostizierten Betroffenen bis zur syndromgerechten Behandlung bei bekannter GCA.

## Syndromspezifische Kurzprofile

### Klinefelter-Syndrom (47,XXY)

Das KS kennzeichnet ein zusätzliches X‑Chromosom bei männlichem Phänotyp. Etwa 87 % weisen den 47,XXY-Karyotyp auf, wobei auch Mosaike (Mischung von Zelllinien mit unterschiedlichem Chromosomensatz) oder höhergradige Aneuploidien vorkommen [2].

**Abb. 1:**
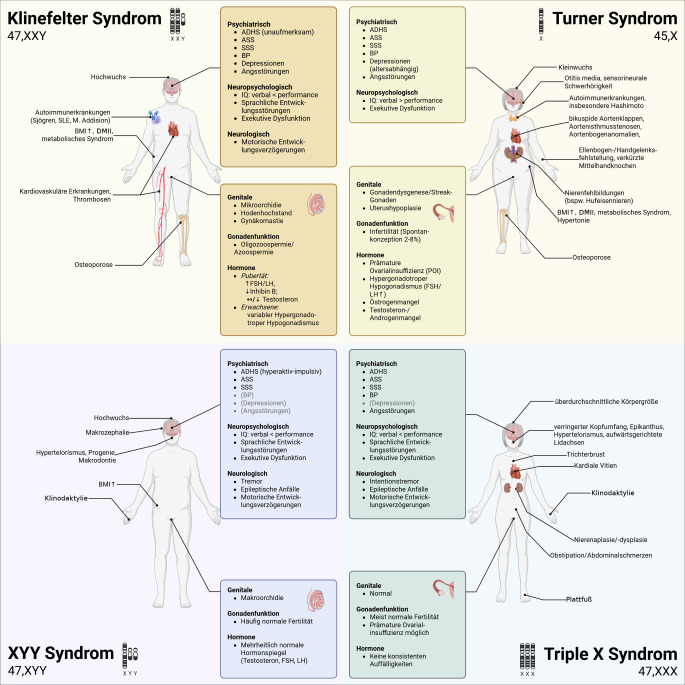
Variabel ausgeprägte Merkmale geschlechtschromosomaler Aberrationen. (Erstellt mit BioRender. Paland H. 2026; https://BioRender.com/y8z9ygi.) *ADHS *Aufmerksamkeitsdefizit‑/Hyperaktivitätsstörung,* ASS *Autismusspektrumstörungen,* BMI *Body-Mass-Index, *BP *bipolare affektive Störungen, *DMII *Diabetes mellitus Typ 2, *FSH *follikelstimulierendes Hormon,* IQ *Intelligenzquotient,* LH *luteinisierendes Hormon, *POI *prämature Ovarialinsuffizienz, *SLE *systemischer Lupus erythematodes,* SSS* Schizophreniespektrumstörungen

Der somatische Phänotyp ist durch Hochwuchs, Mikroorchidie mit Hypogonadismus mit konsekutiver Infertilität durch Azoospermie geprägt [15]. Auch Gynäkomastie kann variabel auftreten, wobei die Diagnosestellung des KS häufig erst bei Fertilitätsabklärungen erfolgt [2, 25]. Hinzu kommen häufig eine weibliche Körperfettverteilung sowie ein erhöhtes Risiko für metabolische Syndrome, Typ-2-Diabetes, Osteoporose, kardiovaskuläre Erkrankungen und thromboembolische Ereignisse im Erwachsenenalter [1, 15]. Autoimmunerkrankungen treten gehäuft auf, wobei das deutlich erhöhte relative Risiko (RR) für Sjögren-Syndrom (RR = 19,3), systemischen Lupus erythematodes (RR = 18,1) und Morbus Addison (RR = 11,7) herausstechen [24]. Bei etwa der Hälfte kommt es während der Pubertät zu hypergonadotropem Hypogonadismus, sodass eine Testosteronsubstitution erforderlich wird (Abb. 1; [15, 25]).

### (Ullrich‑)Turner-Syndrom (45,X)

Das TS betrifft phänotypische Frauen mit partiell oder vollständig fehlendem X‑Chromosom, wobei etwa 40–50 % die klassische 45,X-Monosomie zeigen [10]. Dabei zeigt sich eine Tendenz milderer phänotypischer Merkmale des TS bei nichtklassischen Karyotypen [10] und einer höheren Morbidität und Mortalität bei klassischen 45,X [10].

Der somatische Phänotyp des TS wird durch Kleinwuchs als Kardinalmerkmal charakterisiert, wobei die Endgröße unbehandelt etwa 20 cm unter der Zielgröße liegt [10]. Gonadendysgenesie mit konsekutivem Hypogonadismus und primärer Amenorrhö stellen ein weiteres Kernmerkmal des TS dar [10]. Eine spontane Pubertätsentwicklung findet nur bei etwa einem Fünftel statt (niedrige Raten bei 45,X, höhere Raten bei Mosaiken; [5]). Wachstumshormontherapie zur Verbesserung der Endgröße sowie Östrogengabe zur Pubertätsinduktion werden daher häufig genutzt [8].

Kardiovaskuläre Anomalien können auftreten (bikuspide Aortenklappen, Aortenisthmusstenosen und Aortenbogenanomalien; [10]). Hypertonie tritt bei über der Hälfte der Erwachsenen auf, das Schlaganfallrisiko ist nahezu verdreifacht [10]. Weitere Manifestationen umfassen erhöhte metabolische Risiken für bspw. Diabetes und Osteoporose. Autoimmunerkrankungen treten gehäuft auf, wobei insbesondere Schilddrüsenerkrankungen bis zu 50 % der Patientinnen betreffen. Zumeist asymptomatische Nierenfehlbildungen wie Hufeisennieren finden sich bei 24–42 % [10]. Gehäufte kindliche Otitis media und sensorineurale Schwerhörigkeit im Erwachsenenalter sind außerdem zu beobachten (Abb. 1; [10]).

### XYY-Syndrom (47,XYY)

Das XYY ist durch das Vorhandensein eines zusätzlichen Y‑Chromosoms bei männlichem Phänotyp charakterisiert. Im Gegensatz zu anderen GCA ist der somatische Phänotyp häufig unauffällig, was eine der Hauptursachen für die späte oder fehlende Diagnosestellung darstellt [2, 15].

Somatisch beobachtet werden kann ein Hochwuchs, der ähnlich ausgeprägt ist wie beim KS [25]. Weitere körperliche Merkmale können diskrete Dysmorphien wie Makrozephalie, orbitaler Hypertelorismus und erhöhtes Hodenvolumen umfassen [15]. Im deutlichen Gegensatz zum KS sind Testosteronspiegel, Gonadotropinfunktion und Fertilität meist normal (Abb. 1; [15, 25]).

### Triple-X-Syndrom (47,XXX)

Das XXX liegt bei Trägerinnen eines zusätzlichen X‑Chromosoms vor. Die körperlichen Merkmale zeigen sich hier ebenso diskret und unspezifisch, sodass sie selten unmittelbar Anlass für eine genetische Testung geben [25].

Die möglichen somatischen Merkmale des XXX umfassen überdurchschnittliche Körpergröße, Epikanthusfalten, Klinodaktylie und Hypotonie [25, 28]. Im Gegensatz zum TS ist die Fertilität meist normal, obwohl vorzeitiges Ovarialversagen auftreten kann [25]. Urogenitale Anomalien und epileptische Anfälle wurden in einigen Studien für XXX berichtet (Abb. 1; [25]).

In etwa 10 % der Fälle liegen Mosaike vor, mit verschiedenen Kombinationen wie 46,XX/47,XXX oder 45,X/47,XXX bzw. 45,X/46,XX/47,XXX [25, 28]. Höhergradige Polysomien wie 48,XXXX und 49,XXXXX sind selten, aber mit schwereren Verläufen assoziiert [19].

## Epidemiologie

In Tab. 1 sind die epidemiologischen Kennzahlen der vier häufigsten GCA basierend auf Registerdaten zusammengefasst [1, 2, 23]. Vergleichbare deutsche Daten existieren nicht. Die erwarteten biologischen Prävalenzen sind populationsübergreifend konsistent [1], während die Detektionsraten von lokaler diagnostischer Infrastruktur und klinischer Aufmerksamkeit abhängen und auch innerhalb Europas erheblich variieren [1].

Die Detektionsraten zeigen ein heterogenes Bild: Während TS fast vollständig erkannt wird, liegen die Raten für KS, XXX und XYY deutlich niedriger [2, 23]. Diese Unterschiede spiegeln am ehesten das Fehlen spezifischer somatischer Leitsymptome wider [2, 25]. Das KS kann im Erwachsenenalter noch durch die häufige Infertilität auffallen, während bei XXX und XYY ein solcher diagnostischer Trigger fehlt. Das niedrigere mediane Diagnosealter bei XXX und XYY dürfte daher weniger eine frühere Manifestation als vielmehr das Fehlen nichtdiagnostizierter Erwachsener in den Registern widerspiegeln [1, 2].

**Tab. 1:** Epidemiologie geschlechtschromosomaler Aberrationen. (Prävalenz und Diagnosealter nach [1]; Detektionsraten nach [2, 21])

	TS	XXX	KS	XYY
Erwartete Prävalenz (pro 100.000)	50	84	152	98
Detektionsrate (%)	70–93	7–15	22–23	9–15
Medianes Diagnosealter (Jahre)	15,1	17,9	27,5	15,1

*KS* Klinefelter-Syndrom, *TS* Turner-Syndrom

## Psychiatrische Manifestationen

Geschlechtschromosomale Aberrationen zeigen substanziell erhöhte Risiken für psychiatrische Symptomatik mit einer Hazard Ratio (HR) von 2,20 bis 4,30, wobei ADHS, ASS, SSS, Angst- und affektive Störungen die häufigsten Diagnosen darstellen (Tab. 2).

**Tab. 2:** Risiko für psychiatrische Manifestationen bei geschlechtschromosomalen Aberrationen

Störung	TS	XXX	KS	XYY
ADHS	↑↑↑^a^	↑^a^–↑↑↑^b^	↑^a^	↑↑^a^
ASS	↑↑↑^a,b,c^	↑↑↑^b^	↑^a^–↑↑↑^c^	↑↑↑^a^
Angststörung	(↑)	↑↑^b,d^	(↑)^d^	(↑)^d^
Depression	(↑)^*,a^	(↑)^a,b,d,e^	(↑)^a,d^	↑^a,d^
SSS	(↑)^a,c^	↑↑^a,e^	↑↑^a,c^	↑↑^a^
BP	(?)	↑↑^a^	↑↑^c^	(↑)^a^

*ADHS *Aufmerksamkeitsdefizit‑/Hyperaktivitätsstörung,* ASS *Autismusspektrumstörungen, *BP *bipolare affektive Störungen, *KS* Klinefelter-Syndrom, *TS* Turner-Syndrom, *SSS* Schizophreniespektrumstörungen

Risiko Hazard Ratio/Odds Ratio: ↑ = 2–3, ↑↑ = 3–5, ↑↑↑ = > 5, (↑/?) = inkonsistente/fehlende Datenlage; * = altersabhängig, v. a. Erwachsenenalter. Datenquellen finden sich nachfolgend bzw. als Einzelbelege im Text:

^a^ Bevölkerungsbasiert, mittleres Alter 10,9 Jahre, max. 35 Jahre [23]

^b^ Pädiatrische Registerdaten, < 18 Jahre [14, 17]

^c^ Registerbasiert, alle Altersgruppen ohne Aufschlüsselung der Altersstruktur [3, 4]

^d^ Adulte Biobankdaten, 55–68 Jahre [6]

^e^ Adulte Kohortendaten, mittleres Alter 33 Jahre [21]

Wesentliche Daten zu psychiatrischen Manifestationen, die im Weiteren beschrieben werden, stammen aus bevölkerungsbasierten Registern und spezialisierten Klinikkohorten. Die dänische iPSYCH2015-Studie umfasst genotypisierte Personen im Alter von 0 bis 34,7 Jahren (mittleres Alter 10,9 Jahre, Standardabweichung ±3,5 Jahre, *n* = 78.726 Menschen mit einer psychiatrischen Diagnose, *n* = 43.326 Vergleichskohorte; [23]). Die schwedischen Registerdaten erfassen landesweite Diagnosen über die gesamte Lebensspanne für TS [3] und KS [4]. PEDSnet (USA) umfasst Gesundheitsdaten von ca. 6 Mio. pädiatrischen Fällen (< 18 Jahre) aus sechs akademischen Zentren [14, 17]. Die eXtraordinarY Kids Clinic (USA) ist eine spezialisierte interdisziplinäre Sprechstunde mit über 400 betreuten Kindern und Jugendlichen (6–20 Jahre) mit GCA [26, 27]. Für das Erwachsenenalter liegen durch eine phänotypweite Assoziationsstudie (PheWAS) Daten von 1,5 Mio. genotypisierten Teilnehmern dreier Biobanken (Alter zur letzten Erhebung 55–68 Jahre) für KS, XXX und XYY vor [6]. Dies wird ergänzt durch strukturierte psychiatrische Interviews in einer spezialisierten XXX-Kohorte (*n* = 34, mittleres Alter 32,9 Jahre, SD 13,1 Jahre; [21]). Insgesamt erhöhte Prävalenzraten von GCA bei psychiatrischen Erkrankungen ließen sich anhand der iPSYCH2015-Daten berechnen (vgl. Tabelle S1). Eine direkte Altersstratifizierung der Risikokennziffern ist anhand der vorliegenden Studien nicht möglich, da keine der Primärquellen altersgestufte Prävalenzschätzer berichtet.

### Aufmerksamkeitsdefizit‑/Hyperaktivitätsstörung

Die Aufmerksamkeitsdefizit‑/Hyperaktivitätsstörung stellt eine der häufigsten psychiatrischen Manifestationen bei GCA dar. Beim *Klinefelter-Syndrom* erfüllen 34–63 % der Kinder und Jugendlichen die Kriterien nach DSM-IV(Diagnostic and Statistical Manual of Mental Disorders IV) einer ADHS, wobei beim KS Symptome der Unaufmerksamkeit dominieren [15, 29]. Das allgemeine ADHS-Risiko beim KS ist signifikant erhöht (HR 1,99, 95 %-[Konfidenzintervall]KI 1,24–3,19; [23]).

Bei etwa der Hälfte der *Turner-Syndrom-Betroffenen* manifestieren sich ADHS-typische Eigenschaften [11]. Schwedische Registerdaten zeigen interessanterweise geringe ADHS-Diagnoseraten, was dafür sprechen könnte, dass exekutive Dysfunktionen als syndrominhärente Merkmale nicht eigenständig diagnostiziert werden [3]. Andere Studien zeigen robust ein deutlich erhöhtes ADHS-Risiko beim TS (HR 6,15, 95 %-KI 1,63–23,19; [23]).

Bei *XYY* wird ADHS mit Prävalenzen von 11–52 % dokumentiert, wobei anteilige Symptome bei bis zu 76 % zu finden sind [2, 29]. Im Gegensatz zum KS zeigen sich bei XYY häufiger Symptome des hyperaktiv-impulsiven Typs [27]. Das Risiko für ADHS ist bei XYY ebenso signifikant erhöht (HR 4,45, 95 %-KI 2,56–7,72; [23]).

Bei *XXX* findet sich ADHS bei 25–49 % der Patientinnen, wobei der unaufmerksame Subtyp dominiert [25, 29]. Angaben zu Risikokennziffern variieren etwas, sind aber insgesamt erhöht (HR 2,67, 95 %-KI 1,11–6,41, [23]; Odds Ratio [OR] 7,0, 95 %-KI 4,3–11,3, [14]).

Die Aufmerksamkeitsdefizit‑/Hyperaktivitätsstörung ist mit Prävalenzen von 25–63 % eine auffallend häufige psychiatrische Manifestation bei GCA, wobei bei KS und Trisomie X der unaufmerksame, beim XYY der hyperaktiv-impulsive Subtyp dominiert. In adulten Biobankdaten zeigte sich interessanterweise keine signifikante ADHS-Assoziation bei den drei Trisomien (KS, XYY, XXX; [6]), was am ehesten auf die in diesen Geburtskohorten kaum etablierte Erwachsenendiagnostik zurückzuführen sein dürfte.

### Autismusspektrumstörung

Autismusspektrumstörungen treten bei GCA deutlich gehäuft auf. Beim *Klinefelter-Syndrom* ist die HR auf 2,72 erhöht (95 %-KI 1,72–4,32; [23]). Schwedische Registerdaten zeigten für KS ein noch stärker erhöhtes Risiko (OR 6,2, 95 %-KI 4,0–9,4) mit einer ASS-Prävalenz von 2,8 % (vs. 0,5 % der Kontrollstichprobe; [4]). Dokumentierte Prävalenzen beim KS zeigen eine methodenabhängige Variabilität. Fragebogenbasierte Screenings ergaben Raten von 11–12 %, während diagnostische Interviews Prävalenzen zwischen 5 % (ADOS) und 27 % (ADI-R) zeigen [13]. Charakteristische autistische Merkmale beim KS umfassen Defizite in der Wahrnehmung sozial-emotionaler Hinweisreize, beeinträchtigtes Emotionsverständnis, Probleme bei der Interpretation affektiver Prosodie und reduzierten Blickkontakt, wobei soziale Zurückhaltung und Kommunikationsschwierigkeiten durch verbale Defizite verstärkt werden [13, 15].

Die Prävalenz von ASS beim *Turner-Syndrom* wird übereinstimmend als erhöht beschrieben. In pädiatrischen Registerdaten wurde eine Prävalenz von 3,4 % berichtet (OR 2,41, 95 %-KI 1,79–3,23; [17]), was gut vereinbar ist mit schwedischen und dänischen Registerdaten (Prävalenz 2,16 %, OR 4,26, 95 %-KI 2,94–6,18, [3]; HR 8,45, 95 %-KI 2,49–28,61, [23]).

Für *XYY* muss mit durchschnittlichen Prävalenzen von 30 % eine noch stärker ausgeprägte Problematik angenommen werden [29]. Das Risiko bei XYY ist deutlich erhöht mit einer HR von 5,61 (95 %-KI 3,35–9,40; [23]). Einige Screeningstudien zeigten sogar, dass etwa 50 % der Jungen mit XYY (vs. 12 % bei KS) Werte im Bereich eines möglichen Autismus erreichen [13].

Beim *XXX* zeigt sich ebenso eine gesteigerte Symptomprävalenz von etwa 11–20 % [29] und ein relativ stark erhöhtes Risiko (OR 7,7, 95 %-KI 3,9–15,3; [14]).

### Angststörung

Bei durchschnittlich 26 % (14–27 %) der *Klinefelter-Syndrom-Betroffenen* werden Angststörungen beobachtet, gegenüber unabhängig berichteten Prävalenzen von 7 % der Allgemeinbevölkerung [29]. Charakteristisch sind beim KS insbesondere soziale Ängstlichkeit und soziale Zurückhaltung [13, 29]. Zudem bestehen Schwierigkeiten beim Erkennen und Verbalisieren eigener Emotionen, was die Implementierung von Emotionsregulationsstrategien erschwert [29]. Im Erwachsenenbereich stützen populationsbasierte Biobankdaten eine moderate Risikoerhöhung (OR 1,8, 95 %-KI 1,6–2,1; [6]).

Beim *Turner-Syndrom* werden hohe Lebenszeitprävalenzen von bis zu 52 % angegeben, wobei ein breites Spektrum in Form generalisierter Angst, spezifischer Phobien und vor allem sozialer Ängste anzutreffen ist [16]. Neuroimaging-Studien bei TS zeigen u. a. vergrößerte Amygdalae, was als plausibles neurobiologisches Korrelat der Angststörungen gewertet werden kann [12].

Beim *XYY* sind Angststörungen weniger gut untersucht. Teils werden unauffällige Raten von Angst berichtet, aber einzelne Studien zeigten eine Rate von 26 % bei XYY (vs. 7 % der Allgemeinbevölkerung) mit Angststörung [13, 29]. In adulten Biobankdaten war die Assoziation gering (OR 1,2, 95 %-KI 1,1–1,4; [6]).

Etwa 20 % der *XXX-Patientinnen* leiden an Angststörungen [29]. Einzelne Angstsymptome treten jedoch insgesamt häufiger auf, z. B. in Form von sozialer Ängstlichkeit, generalisierter Angst und Trennungsangst [25, 29]. Registerdaten zeigen passend dazu ein erhöhtes Risiko für Angststörungen (OR 4,2, 95 %-KI 2,8–6,2; [14]). Adulte Biobankdaten bestätigen ein erhöhtes Risiko (OR 2,2, 95 %-KI 1,6–3,0; [6]).

### Depression

Beim *Klinefelter-Syndrom* berichten Studien für Depressionen durchschnittlich Diagnoseraten von 27 % (12–68 %; [29]), passend zum signifikant erhöhten Risiko (HR 1,88, 95 %-KI 1,07–3,33; [13, 23]) im Einklang mit adulten Biobankdaten für affektive Störungen (OR 1,9, 95 %-KI 1,6–2,1; [6]).

Für das *Turner-Syndrom* findet sich in dänischen Registerdaten jüngerer Teilnehmer keine erhöhte Risikokonstellation [23]. Allerdings zeigt eine systematische Übersichtsarbeit über 35 Studien, dass depressive Episoden beim TS altersabhängig besonders im Erwachsenenalter gehäuft auftreten [20]. Die erhöhte Vulnerabilität bei Erwachsenen könnte auf die psychosoziale Auseinandersetzung mit Infertilität, körperlichen Besonderheiten und verzögerter Pubertätsentwicklung sowie auf hormonelle Faktoren und assoziierte sozialkognitive Schwierigkeiten zurückgeführt werden [16, 20].

Beim *XYY* sind depressive Symptome ebenso weniger gut untersucht und werden z. B. mit einer Rate von 13 % angegeben [13, 29], wobei das Risiko moderat erhöht ist (HR 2,65, 95 %-KI 1,12–5,90; [23]), was sich auch in der Erwachsenenpopulation in Biobankdaten für affektive Störungen zeigt (OR 1,4, 95 %-KI 1,2–2,5; [6]). Die Risikosteigerung gegenüber Menschen ohne XYY war in pädiatrischen Registerdaten nicht zu finden [14], ob analog zum TS ein altersabhängiger Effekt besteht, ist noch unklar.

Für *XXX* werden Raten diagnostizierter Depressionen zwischen 18–54 % berichtet [29]. In einer erwachsenen XXX-Kohorte lag die Lebenszeitprävalenz für major-depressive Episoden bei 43,3 % (Cramér’s V = −0,34, *p* = 0,011; [21]). Während einige Registerdaten [14, 23] keine erhöhten Risiken bei XXX zeigen, findet sich in adulten Biobankdaten ein moderat erhöhtes Risiko für affektive Störungen bei XXX (OR 1,9, 95 %-KI 1,4–2,5; [6]).

### Schizophreniespektrumstörungen und bipolare affektive Störung

Beim *Klinefelter-Sxndrom* belegen Studien erhöhte Risiken für psychotische und bipolare affektive Störungen (BP). Das Risiko für SSS ist etwa dreifach erhöht (HR 2,92, 95 %-KI 1,60–5,35, [23]; OR 3,6, 95 %-KI 2,0–6,7 [4]). Cederlöf et al. beziffern das Risiko für nach DSM-IV diagnostizierte BP bei KS auf eine OR von 3,8 (95 %-KI 1,8–7,6; [4]).

In jüngeren Studien zum *Turner-Syndrom* zeigt sich auch für SSS ein etwa verdoppeltes Risiko (OR 1,98, 95 %-KI 1,36–2,88, [3]; HR 1,80, 95 %-KI 1,15–2,80 [23]).

Bei *XYY* besteht ein erhöhtes Risiko für SSS (HR 4,60, 95 %-KI 1,57–13,51), aber kein signifikantes Risiko für BP [23], wobei an anderer Stelle dennoch eine Rate von 8 % BP bei XYY berichtet wird [29].

Das *XXX* zeigt erhöhte Risiken sowohl für SSS (HR 4,54, 95 %-KI 1,58–12,98) als auch für BP (HR 4,32, 95 %-KI 1,12–16,62; [23]). In einer erwachsenen XXX-Kohorte lag die Lebenszeitprävalenz psychotischer Störungen bei 29,4 % (Cramér’s V = −0,41, *p* = 0,001; [21]).

Das volle Ausmaß psychiatrischer Manifestationen ist wegen der hohen Unterdiagnostik und des systematischen Erfassungsbias wahrscheinlich noch nicht vollständig verstanden. Die bisherigen Daten deuten aber deutlich in Richtung erhöhter Prävalenzen (schwerer) psychiatrischer Symptome.

## Klinische Implikationen

### Diagnostische Strategien

Diagnostische genetische Untersuchungen können in Deutschland von approbierten Ärzten veranlasst werden (prädiktive Testungen bei nichtsymptomatischen Personen nur mit Zusatzqualifikation) und sind im Krankenversicherungssystem extrabudgetär. Benötigt wird Heparin- oder EDTA(Ethylendiamintetraessigsäure)-Blut, wobei eine Abstimmung mit dem durchführenden humangenetischen Labor ratsam ist. Die Wahl der geeigneten Methodik (z. B. Karyotypisierung) erfolgt durch die humangenetischen Ärzte des Labors, zur Anforderung muss ärztlich lediglich die Indikation zur genetischen Abklärung gestellt werden. Bei auffälligen Befunden ist eine genetische Beratung anzubieten. Bei Bedarf ist auch eine Vermittlung an eine Spezialambulanz für Seltene Psychische Erkrankungen oder Genetik Psychischer Erkrankungen möglich (z. B. an den Universitätsklinika in Bonn, Hamburg, Hannover oder Würzburg).

Für die psychiatrische Praxis stellt sich die Frage, in welchen Fällen eine bislang undiagnostizierte GCA in Betracht gezogen und eine genetische Testung veranlasst werden sollte. Aktuelle Empfehlungen zur genetischen Diagnostik bei psychischen Erkrankungen formulieren übereinstimmend klinische Konstellationen, die eine genetische Abklärung nahelegen [7, 18, 22]. Hierzu zählen atypische Krankheitsverläufe, Behandlungsresistenz, früher Krankheitsbeginn oder ungewöhnliche Schwere, die Kombination psychiatrischer Symptome mit kognitiven Auffälligkeiten oder Intelligenzminderung, neurodevelopmentale Komorbiditäten wie ADHS oder ASS, anamnestische Hinweise auf Entwicklungsstörungen sowie die Koexistenz psychischer Erkrankungen mit somatischen Befunden wie Organfehlbildungen, Dysmorphiezeichen oder auffälligen Körpermaßen [7, 18, 22].

Aus den allgemeinen Empfehlungen leiten sich für GCA spezifische Indikationskriterien ab. In der psychiatrischen Praxis sollte eine genetische Diagnostik insbesondere erwogen werden, wenn psychiatrische Symptome mit somatischen Auffälligkeiten wie Hochwuchs, Kleinwuchs, Infertilität, Hypogonadismus oder kongenitalen Anomalien einhergehen [7, 18, 22]. Weitere Hinweise sind eine Diskrepanz zwischen kognitivem Leistungsniveau und familiärem Bildungshintergrund, Körpermaße oberhalb der 97. oder unterhalb der 3. Perzentile sowie therapieresistente oder ungewöhnlich früh manifestierende Verläufe bei gleichzeitiger neurodevelopmentaler Komorbidität [7, 18]. Zwar können syndromspezifische somatische Merkmale richtungsweisend sein (Abb. 1), doch riskiert eine ausschließliche Orientierung an körperlichen Auffälligkeiten fortgesetzte Unterdiagnostik bei diskreten oder unauffälligen Phänotypen. Entscheidend ist daher die Zusammenschau aus detaillierter Anamnese in Verbindung mit psychiatrischen und somatischen Befunden im Sinne eines klinischen Gesamteindrucks. Zur Unterstützung dieser Einschätzung haben wir eine klinische Handlungsempfehlung formuliert (vgl. Supplement 1), die auf den genannten Indikationskriterien basiert, jedoch bislang nicht wissenschaftlich validiert wurde.

### Therapeutische Ansätze

Bis dato existieren keine GCA-spezifischen Studien zur Psychopharmakotherapie bei Erwachsenen. Die Behandlung folgt den üblichen Leitlinienempfehlungen, wobei syndromspezifische somatische Risiken zu beachten sind. Beim TS sollte eine mögliche QTc-Verlängerung unter Medikation genauer kontrolliert werden [10]. Beim KS gilt es, das erhöhte Risiko für metabolisches Syndrom bei Antipsychotika zu berücksichtigen. Für KS, XYY und XXX besteht ein erhöhtes venöses Thromboembolierisiko (OR 4,1–8,1; [6]). Für die ADHS-Behandlung bei GCA zeigen retrospektive Daten Stimulanzienansprechraten von ca. 79 %, vergleichbar mit der Allgemeinbevölkerung [27]. Hormonsubstitution ist häufig indiziert (Testosteron beim KS, Östrogen lebenslang beim TS [10]) und kann Stimmung, Antrieb und metabolisches Profil günstig beeinflussen, wobei direkte Effekte auf psychiatrische Symptome nicht gut untersucht sind [10, 13]. Eine endokrinologische Anbindung wird für alle GCA empfohlen [9].

Betroffene zeigen eine erhöhte psychosoziale Belastung und Gesamtmortalität bei hoher Rate psychiatrischer Unterversorgung [1]. Spezialisierte interdisziplinäre Versorgungsstrukturen fehlen weitgehend. *Der kollegiale Austausch über Fachgrenzen hinweg und der Aufbau eigener lokaler Netzwerke sind daher ein pragmatischer erster Schritt.*

## Diskussion und Limitationen

Die präsentierte Evidenzlage ist heterogen. Klinische Kohorten überrepräsentieren schwer Betroffene, populationsbasierte Studien zeigen teils niedrigere Risikoschätzer. Die Hauptdatenquellen unterscheiden sich erheblich in Altersstruktur und Studiendesign, was die Vergleichbarkeit einschränkt. Spät manifestierende Störungen werden in jüngeren Kohorten systematisch unterschätzt. Dennoch bestätigen unabhängige Datenquellen übereinstimmend ein erhöhtes Risiko psychiatrischer Manifestationen bei allen vier GCA. Für Deutschland fehlen populationsbasierte Daten. Längsschnittstudien und Interventionsstudien mit GCA-spezifischen Endpunkten bleiben ein Desiderat.

## Fazit für die Praxis

- Bei der Kombination psychiatrischer Symptome mit somatischen Auffälligkeiten oder atypischen Verläufen sollte an die Differenzialdiagnose einer geschlechtschromosomalen Aberration (GCA) gedacht werden.
- Eine genetische Diagnostik kann niederschwellig direkt von jedem approbierten Arzt veranlasst werden.
- Jede molekulare Diagnose einer GCA bietet eine Chance für eine gezielte Intervention und verbesserte Langzeitprognose.

## Supplementary Information

ESM 1: Klinische Handlungsempfehlung: Wann sollte in der psychiatrischen Praxis an eine geschlechtschromosomale Aberration (GCA) gedacht werden?

ESM 2: Tabelle S1 Geschätzte relative Prävalenz geschlechtschromosomaler Aberrationen bei psychiatrischen Manifestationen

## Data Availability

Alle dieser Arbeit zugrunde liegenden Daten sind in diesem Artikel oder in den Referenzen enthalten.
