# Supplementary material for: Neuropsychiatric manifestations of sex chromosome aberrations—Clinical and therapeutic aspects of neuropsychiatric care
Source: Nervenarzt. 2026 Jun 2;97(4):326–38. [Article in German] doi: 10.1007/s00115-026-01977-0 (PMC13315307; doi:10.1007/s00115-026-01977-0)
Supplement: Supplementary file 1 — ESM 1: Klinische Handlungsempfehlung: Wann sollte in der psychiatrischen Praxis an eine geschlechtschromosomale Aberration (GCA) gedacht werden? [file 115_2026_1977_MOESM1_ESM.docx]

**Klinische Handlungsempfehlung: Wann sollte in der psychiatrischen Praxis an eine Geschlechtschromosomale Aberration (GCA) gedacht werden?**

**Wichtiger Hinweis:** Die nachfolgenden Empfehlungen sind nicht als validiertes Screening-Instrument zu verstehen, sondern als klinische Orientierungshilfe. Es handelt sich explizit nicht um eine Richt- oder Leitlinie.

**Abkürzungen:** ADHS: Aufmerksamkeitsdefizit-/Hyperaktivitätsstörung; ASS: Autismus-Spektrum-Störung; GCA: geschlechtschromosomale Aberration; GenDG: Gendiagnostikgesetz; KS: Klinefelter-Syndrom (47,XXY); KV: Kassenärztliche Vereinigung; SLE: Systemischer Lupus erythematodes; TS: Turner-Syndrom (45,X); VTE: venöse Thromboembolie; XXX: Triple-X-Syndrom (47,XXX); XYY: XYY-Syndrom (47,XYY)

# Allgemeine Indikationsprinzipien und ihre Anwendung auf GCA

Da bis zu 86 % der GCA-Betroffenen nicht diagnostiziert sind [2], ist davon auszugehen, dass psychiatrische Praxen bereits Betroffene behandeln, ohne die genetische Grundlage zu kennen. Das Royal College of Psychiatrists benennt GCA explizit als Differenzialdiagnose [7]. Kilarski et al. [6] haben acht klinische Konstellationen formuliert, bei denen eine genetische Diagnostik erwogen werden sollte. Die folgenden Kriterien leiten daraus GCA-spezifische Anwendungen ab [3, 6, 7]. Keines ist für sich allein spezifisch; je mehr Bereiche jedoch gleichzeitig betroffen sind, desto eher sollte eine genetische Diagnostik erwogen werden.

**Atypischer Verlauf:** Behandlungsresistenz, früher Krankheitsbeginn oder schwerer Verlauf können auf eine genetische Grundlage hinweisen [6]. Bei GCA können kognitive Besonderheiten (z. B. Diskrepanz zwischen verbalem und Handlungs-IQ beim KS, visuell-räumliche Defizite beim TS) die Psychotherapie-Wirksamkeit und hormonelle Faktoren (Hypogonadismus) affektive Symptome beeinflussen [5, 8]. Bei therapieresistenten affektiven Störungen kann die Bestimmung von Testosteron bzw. Östradiol differenzialdiagnostisch aufschlussreich sein.

**Familienanamnese:** GCA entstehen überwiegend *de novo* durch meiotische Non-Disjunction [5, 8]. Eine fehlende psychiatrische Familienbelastung bei schwerer Symptomatik kann daher die Vortestwahrscheinlichkeit für eine seltene genetische Ursache, darunter GCA, erhöhen.

**Multisystemische Symptome:** Gleichzeitige Behandlung in mehreren Fachdisziplinen ohne ätiologische Zusammenführung kann auf eine GCA hinweisen [6]. Da alle drei Trisomien ein 4- bis 8-fach erhöhtes VTE-Risiko aufweisen [2], hat auch eine VTE-Anamnese differenzialdiagnostische Relevanz.

**Kognitive Auffälligkeiten:** Bei GCA liegt meist keine klassische Intelligenzminderung vor, aber charakteristische kognitive Profile [5, 8]. Die Diskrepanz zwischen kognitiver Leistung und familiärem Bildungshintergrund ist diagnostisch relevanter als der absolute IQ [3].

**Entwicklungsstörungen in der Anamnese:** Sprachentwicklungsstörungen, motorische Verzögerungen und schulische Schwierigkeiten finden sich bei allen vier GCA regelhaft [5, 8]. Die gezielte Frage nach Frühförderung (Logopädie, Ergotherapie) kann in der Erwachsenenpsychiatrie den entscheidenden Hinweis liefern [3, 6].

**Kombination multipler psychiatrischer Diagnosen:** Autismus-Spektrum-Störungen (ASS) oder Aufmerksamkeitsdefizit-/Hyperaktivitätsstörungen (ADHS) allein rechtfertigen keine routinemäßige genetische Testung, wohl aber in Kombination mit zusätzlichen psychiatrischen, somatischen oder entwicklungsanamnestischen Auffälligkeiten [6, 7]. ASS-Raten bei GCA liegen zwischen 2,8 % (KS) [1] und 30 % (XYY) [10]. Hellhörig werden sollten Behandelnde auch, wenn Patienten eine Reihe von psychiatrischen Diagnosen bekommen, wie z. B. eine paranoide Schizophrenie und eine ADHS oder in der Kindheit eine ASS gefolgt von einer Bipolar Diagnose im Erwachsenenalter, da dies häufig Ausdruck ungeklärter, aber auffälliger klinisch-psychiatrischer Manifestationen ist.

**Somatische Komorbiditäten:** Richtungsweisend können sein: Hoch- oder Kleinwuchs, Hypogonadismus, Infertilität, Autoimmunerkrankungen (Hashimoto-Thyreoiditis, systemischer Lupus erythematodes), venöse Thromboembolien [2] und metabolisches Syndrom. Gezielte somatische Suchfragen (Körpergröße, Pubertätsentwicklung, Fertilitätsanamnese, Schilddrüsenstatus) können diagnostisch aufschlussreich sein.

# Klinische Szenarien und Handlungsempfehlungen

Im klinischen Alltag ergeben sich drei grundlegende Konstellationen:

## Szenario 1: GCA ist nicht bekannt, kein klinischer Anhalt

Behandlung störungsspezifisch nach den jeweiligen Leitlinien. Eine genetische Diagnostik ist nicht indiziert.

## Szenario 2: GCA ist nicht bekannt, aber klinische Hinweiszeichen bestehen

Wenn der klinische Gesamteindruck den Verdacht auf eine GCA weckt, sollte genetische Diagnostik erwogen werden. Die Schwelle sollte niedrig angesetzt werden: Die Untersuchung ist minimal-invasiv (venöse Blutentnahme), die Kosten werden im KV-System extrabudgetär getragen [3, 6], und jede approbierte Ärztin/jeder approbierte Arzt kann sie nach Aufklärung und schriftlicher Einwilligung veranlassen (GenDG §7) [3, 6]. Eine Kontaktaufnahme zum jeweiligen humangenetischen Labor im Vorfeld ist ratsam, um logistische Details (Probenversand, Aufklärungsbögen, Laborschein) zu klären. Bei pathologischem Befund ist eine genetische Beratung zu veranlassen (GenDG §10). Die Diagnosestellung kann auch unabhängig von unmittelbaren pharmakologischen Konsequenzen einen psychologischen Benefit darstellen (Psychoedukation, Entstigmatisierung, verbessertes Selbstverständnis) [7].

## Szenario 3: GCA ist bereits bekannt

Bei bekannter GCA sollten Betroffene über das erhöhte Risiko für psychiatrische Manifestationen aufgeklärt und regelmäßig auf häufige Komorbiditäten (insbesondere ADHS, ASS, Angst- und affektive Störungen) gescreent werden. Beim Vorhandensein klinisch relevanter psychischer Symptome sollten diese in Einklang mit den gängigen psychiatrischen Leitlinien behandelt werden. Die im Manuskript benannten Spezifika sind in der Wahl der spezifischen Psychopharmaka zu beachten. Eine interdisziplinäre Versorgung unter Einbindung von Endokrinologie, Humangenetik und je nach klinischer Manifestation weiterer Fachdisziplinen ist anzustreben. Selbsthilfegruppen und syndromspezifische Patientenorganisationen können die Versorgung sinnvoll ergänzen.

# Spezifische Hinweiszeichen

Die folgende Tabelle fasst die klinischen Konstellationen zusammen, bei denen differenzialdiagnostisch an die jeweilige GCA gedacht werden sollte. Die Hinweiszeichen leiten sich aus den in der Übersichtsarbeit dargestellten erkrankungstypischen Profilen ab und sind nicht als validierte Screening-Kriterien zu verstehen.

Tabelle S2 Syndromspezifische Hinweiszeichen

| Klinische Konstellation | Besonders denken an | Schlüsselfragen in der Anamnese | Quellen |
| --- | --- | --- | --- |
| Psychiatrische Störung + Kleinwuchs bei weiblicher Patientin | TS | Pubertätsentwicklung spontan? Menstruation? Herzfehler bekannt? Hörprobleme? | [4, 5] |
| Psychiatrische Störung + Hypogonadismus / Infertilität / Gynäkomastie | KS | Kinderwunsch? Azoospermie? Testosteronwerte? Libido? Knochendichte? | [5, 8] |
| Psychiatrische Störung + Hochwuchs bei weiblicher Patientin | XXX | Körpergröße? Zyklusanamnese? Epilepsie? Nierenfunktion? | [5, 8] |
| Psychiatrische Störung + primäre Amenorrhoe / verzögerte Pubertät / vorzeitiges Ovarialversagen | TS, XXX | Menarche wann? Regelmäßige Zyklen? Hormonstatus? | [4, 5] |
| Psychiatrische Störung + Hochwuchs bei männlichem Phänotyp | KS, XYY | Pubertätsentwicklung? Hodengröße? Fertilität? Stimmbruch? | [5, 8] |
| Psychiatrische Störung + unerklärte venöse Thromboembolie(n) | KS, XYY, XXX | VTE-Anamnese? Gerinnungsdiagnostik? Familiäre Thrombophilie? | [2] |
| Multiple psychiatrische Diagnosen (ADHS + ASS + Angst/Depression) + Sprachentwicklungsstörung in der Anamnese | alle GCA | Logopädie in Kindheit? Ergotherapie? Spät sprechen gelernt? Förderschule? Motorische Entwicklung? | [5, 8, 9] |
| Psychiatrische Multimorbidität + kognitive Leistung deutlich unter familiärem Niveau | alle GCA | Schulabschluss? Berufsausbildung? Bildungsniveau der Eltern? Diskrepanz? | [3, 5] |

# Anmerkungen zu Evidenz und Limitationen

Die hier formulierten Empfehlungen stellen eine klinische Orientierungshilfe dar, keine evidenzbasierte Leitlinie. Die genannten Hinweiszeichen sind nicht prospektiv validiert, die syndromspezifischen Profile entstammen überwiegend klinisch rekrutierten Kohorten mit möglichem Ascertainment Bias [2], und systematische Untersuchungen zur GCA-Prävalenz in psychiatrischen Patientenpopulationen fehlen. Die Datenbasis ist überwiegend skandinavisch und anglo-amerikanisch. Populationsbasierte Daten für Deutschland liegen nicht vor.

Dennoch erscheint eine niedrigschwellige genetische Testung bei klinischem Verdacht angesichts der hohen Unterdiagnostik, der geringen Invasivität, der extrabudgetären Abrechenbarkeit und der potenziell erheblichen therapeutischen Konsequenzen gut begründbar.

# Literatur

1. Cederlöf M, Ohlsson Gotby A, Larsson H et al (2014) Klinefelter syndrome and risk of psychosis, autism and ADHD. J Psychiatr Res. https://doi.org/10.1016/j.jpsychires.2013.10.001

2. Davis SM, Liu A, Teerlink CC et al (2025) Phenome-wide association study of male and female sex chromosome trisomies in 1.5 million participants of MVP, FinnGen, and UK Biobank. Am J Hum Genet. https://doi.org/10.1016/j.ajhg.2025.07.017

3. Degenhardt F, Wohlleber E, Jamra RA, Hebebrand J (2023) Genetische Diagnostik im klinischen Alltag der Kinder- und Jugendpsychiatrie – Indikationen, Rahmenbedingungen, Hürden und Lösungsvorschläge. Z Für Kinder- Jugendpsychiatrie Psychother. https://doi.org/10.1024/1422-4917/a000941

4. Gravholt CH, Viuff M, Just J et al (2023) The Changing Face of Turner Syndrome. Endocr Rev. https://doi.org/10.1210/endrev/bnac016

5. Hong DS, Reiss AL (2014) Cognitive and neurological aspects of sex chromosome aneuploidies. Lancet Neurol. https://doi.org/10.1016/S1474-4422(13)70302-8

6. Kilarski LL, Claus I, Binder EB et al (2024) Genetische Diagnostik bei psychischen Erkrankungen im Erwachsenenalter. Nervenarzt. https://doi.org/10.1007/s00115-024-01737-y

7. Royal College of Psychiatrists (2023) College Report CR237– The role of genetic testing in mental health settings. https://www.rcpsych.ac.uk/docs/default-source/improving-care/better-mh-policy/college-reports/College-report-CR237---Genetic-testing-in-mental-health-settings.pdf. Zugegriffen: 04. Mai 2026

8. Skuse D, Printzlau F, Wolstencroft J (2018) Sex chromosome aneuploidies. In: Geschwind DH, Paulson HL, Klein C (Hrsg) Neurogenetics, Part I. Handbook of Clinical Neurology, Bd 147. Elsevier, Amsterdam, S 355–376. https://doi.org/10.1016/B978-0-444-63233-3.00024-5

9. Tartaglia N, Howell S, Wilson R et al (2015) The eXtraordinarY Kids Clinic: an interdisciplinary model of care for children and adolescents with sex chromosome aneuploidy. J Multidiscip Healthc. https://doi.org/10.2147/JMDH.S80242

10. Van Rijn S (2019) A review of neurocognitive functioning and risk for psychopathology in sex chromosome trisomy (47,XXY, 47,XXX, 47, XYY). Curr Opin Psychiatry. https://doi.org/10.1097/YCO.0000000000000471
