# Supplementary material for: Neuropsychiatric manifestations of sex chromosome aberrations—Clinical and therapeutic aspects of neuropsychiatric care
Source: Nervenarzt. 2026 Jun 2;97(4):326–38. [Article in German] doi: 10.1007/s00115-026-01977-0 (PMC13315307; doi:10.1007/s00115-026-01977-0)
Supplement: Supplementary file 2 — ESM 2: Tabelle S1 Geschätzte relative Prävalenz geschlechtschromosomaler Aberrationen bei psychiatrischen Manifestationen [file 115_2026_1977_MOESM2_ESM.pdf]

Tabelle S1 Geschätzte relative Prävalenz von GCA bei psychiatrischen Manifestationen

Störung	TS	XXX	KS	XYY
**ADHS**	**9,6**	2,1	2,2	**3,9**
**ASS**	**15,7**	**3,9**	2,9	**5,3**
**Major Depression**	1,0*	1,8	2,7	1,9
**SSS**	3,5*	**3,5**	**3,8**	**4,0**
**BP**	11,3*	1,6*	1,4*	2,2*

Prävalenzsteigerungen ≥3× fettgedruckt; = insuffiziente Datenlage. Berechnung basierend auf iPSYCH2015-Daten (Lancet Psychiatry (2023). DOI 10.1016/S2215-0366(23)00004-4). Die iPSYCH2015-Studie verwendet ein fallangereichertes Kohorten-Design, in dem psychiatrische Fälle überrepräsentiert sind. Die dargestellten Werte beruhen auf eigenen Berechnungen unter vereinfachenden Annahmen (bei datenschutzbedingter Angabe von <5 Fällen wurde ein Wert von 2 angenommen) und sind als orientierende Größenordnungen zu verstehen, die die tatsächlichen populationsbasierten Verhältnisse überschätzen können.*
